# Toward patient-centered treatment goals for duchenne muscular dystrophy: insights from the “Your Voice” study

**DOI:** 10.1186/s13023-023-02674-w

**Published:** 2023-04-20

**Authors:** Carolyn E. Schwartz, Skyler Jackson, James Valentine, Natalie Miller, Linda Lowes, Danielle Edwards, Christine McSherry, Dimitrios Savva, Alex Lowe, Jordan McSherry, Patti Engel

**Affiliations:** 1 DeltaQuest Foundation, Inc, 31 Mitchell Road, Concord, MA 01742 USA; 2 Departments of Medicine and Orthopaedic Surgery, Tufts University Medical School, Boston, MA USA; 3 Engage Health, Inc, Eagan, MN USA; 4 Hyman, Phelps & McNamara, P.C, Washington, DC USA; 5 Nationwide Children’s Hospital, Columbus, OH USA; 6 Jett Foundation, Plymouth, MA USA; 7 NewYork-Presbyterian Hospital / Morgan Stanley Children’s Hospital, New York, NY USA

**Keywords:** Duchenne muscular dystrophy, Treatment, Goals, Reasons, Outcomes, Patients, Caregivers, Qualitative, Mixed methods

## Abstract

**Background:**

Patient-centered research has emerged as critically important for understanding the impact of treatments on key stakeholders. The subjective experience of quality of life (QOL) is increasingly recognized as fundamental to delineating treatment goals. The present study utilized content analysis of qualitative data and quantitative analysis to highlight important domains of disease burden and underlying reasons for their importance, and to characterize goals for new treatments for Duchenne Muscular Dystrophy (DMD).

**Results:**

The study sample reflected the perspectives of DMD patients and caregivers representing ambulatory, transitional, and non-ambulatory stages of disability progression (n = 20 per category). Open-ended interviews were content-analyzed and non-parametric statistical tests were used to compare ambulation groups. As patients progressed in disability, the noted DMD burdens reflected some differences in functional areas. While daily functioning and sports/recreation remained the most important priority areas across ambulation groups, “health” became less prominent as the disability progressed from ambulatory to transitional to non-ambulatory phases of disability; whereas relationships became more prominent as one progressed to the non-ambulatory phase from the ambulatory or transitional phases (Kruskall Wallis H = 12.24 and 5.28, p = 0.002 and 0.02, respectively). When asked why their burdens were important to them and how it impacted their or their child’s life, self-esteem/confidence was most important for ambulatory patients, and became less prominent for patients in the transitional and non-ambulatory phases of disability (Kruskall Wallis H = 9.46, p = 0.009). In contrast, independence was less important for ambulatory patients, and became increasing prominent for patients in the transitional and non-ambulatory phases of disability (Kruskall Wallis H = 7.35, p = 0.025). Emotional functioning was most prominent for all ambulation groups on their best and worst days. Goals for new DMD treatments focused on functional goals, general QOL goals, and concerns about safety, ease of use, and effectiveness.

**Conclusion:**

This study provides useful information about treatment goals for DMD from the perspective of patients and their caregivers. It highlights some consistent values across the disability trajectory, as well as introducing an evolution of priorities as the person with DMD becomes more disabled. Results provide a roadmap for patient-centered DMD drug development.

## Introduction

Over the past three decades, patient-centered research has emerged as critically important for understanding the impact of treatments on key stakeholders [1, 2]. Patients have become not only central to outcomes measurement for new treatments [3], but are also increasingly integrated into research teams from their inception, through implementation, analysis, and dissemination of results [4–7]. With this increased focus on the patient’s perspective, concepts deemed relevant have grown in depth and breadth, expanding well beyond objective measurement. The subjective experience of quality of life (QOL) is increasingly recognized as fundamental to treatment outcomes [8, 9]. That QOL means different things to different people along a disability trajectory has led to a substantial body of research on adaptation effects [10, 11], resilience [12, 13], and mediators of treatment burden [13, 14].

Early work in the field of QOL relied on qualitative methods to identify and develop concepts that could then be measured using closed-ended questions that were eminently quantitative [15–17]. As the field of QOL research evolved, researchers increasingly used “mixed methods”, which combined qualitative and quantitative methods to yield novel insights [18]. Such approaches involved content analysis of qualitative data collected via open-ended questions, coding this content with numbers representing different themes, and then using statistical methods to compare groups on these coded themes. Mixed-method research has led to important developments in theory, measurement development, program development and evaluation, and evaluation research [18].

The present study utilizes a mixed-method approach to investigate important domains related to burden of illness, underlying reasons for the impact on patients’ lives, and treatment goals for Duchenne Muscular Dystrophy (DMD). DMD is a genetic disorder characterized by progressive muscle degeneration and weakness caused by an absence of dystrophin, a protein that helps keep muscle cells intact [19]. This progressive, rare, and irreversible neuromuscular disorder occurs primarily in males—1 in 5050 live births [20–22]. Usually diagnosed by age 5, the disorder presents as delayed development that includes motor difficulties [23] and may include cognitive impairment and attention deficit disorders [24]. On average by age 10–12, progressive muscle weakness leads to loss of ambulation, upper-limb function problems, and comorbid conditions such as scoliosis and muscular contractures [23]. By age 15, patients experience increased difficulty breathing and life-threatening heart and lung conditions [25]. DMD patients face profound uncertainty regarding lifespan, typically dying in their 20 s to early 30 s [25], although medical advances [20] have led to longer life expectancies [26].

The present study sought to understand how patient or caregiver goals for DMD treatment vary as a function of the severity of disease progression. Disease progression was characterized in terms of ambulation status to facilitate recruitment across phases of ambulation disability. Nonetheless, the underlying reasons (themes) for this variation will be described, and the relative importance of specific domains will be illustrated comparing Best Days and Worst Days. The impact of this fluidity in goals, reasons, and priorities will be discussed in terms of meaningful treatment goals from the perspective of DMD patients and caregivers.

## Methods

### Study planning and commencement

In 2015, the Jett Foundation^1^ provided a patient-reported outcome report to the Federal Drug Administration (FDA) on patients living with Duchenne who were being treated with eteplirsen to help inform regulatory decision making. Since that time, much has happened in the Duchenne space and, as of 2018, there were 29 ongoing clinical trials studying treatments for Duchenne. In 2017, the Jett Foundation’s Duchenne Biotechnology Council, a group of industry partners and key opinion leaders working in the Duchenne space, identified needs in Duchenne trials, including the need to identify aspects of daily living that are important to patients at every stage of the disease. In late 2017, the research group began planning for a survey that would study the patient experience and identify outcomes that are important to them and identified necessary logistical support and funding mechanisms. In early 2018, the group submitted a meeting request through the Office of Patient Affairs and obtained FDA feedback. The FDA inputs were included in the protocol and after IRB approval, the study commenced.

### Sample

The “Your Voice” study sample was recruited from the Jett Foundation, other DMD-related patient advocacy organizations, and patients who had opted-in to be contacted for research participation through Engage Health’s EnCompass® database.^2^ Participants were recruited using email communication and posts to social media sites. Eligible participants were 18 years of age or older, a parent of a patient younger than 17 years of age or a parent of a patient older than 18 years if the patient was unable to answer for themselves; willing and able to sign consent / assent; a United States resident; and willing to participate in a one-hour interview. Participants had to provide documentation of DMD diagnosis of themselves or their child, for patients and caregivers, respectively. Such documentation included a genetic diagnosis from a relevant testing laboratory, physician-consult notes, school notes describing Individual Education Program accommodations and disease name, or medical record of diagnosis.

### Procedure

The “Your Voice” study design and interview questions were developed in collaboration with key stakeholders, including DMD patients (AL), family caregivers (CM, JM), pharmaceutical researchers (DS, SJ, PE), and clinicians (NM, LL). Participant recruitment was stratified by level of ambulation disability to provide representation for people/caregivers of ambulatory, transitional, and non-ambulatory stages of disability progression. Participants were recruited within stage in cohorts of five (5), and recruitment continued until saturation was deemed met (i.e., no new or important information gleaned from the final-cohort interviews [27, 28]).

Following informed consent from an adult patient or caregiver and, when applicable, assent from a minor child,^3^ confirmation of DMD diagnosis, and group assignment based on ambulation stage, study participants were interviewed by telephone by trained interviewers. The interviews were conducted in English and took approximately 45 min.^4^ Participants were allowed to abstain from answering any question, and were allowed to stop at any time. Data were fully de-identified after collection to ensure confidentiality. An honorarium of $100 was paid for each completed interview, and one interview was allowed, representing each person with DMD. Caregivers were asked to represent their child’s experience.

The interview proceeded in two parts and followed a qualitative method developed to better understand patient/caregiver experience [30]. Participants were first asked open-ended questions (“un-aided questions”) about burdens associated with DMD, including important functions which DMD prevented the individual from doing, and why it was important to them. The interview utilized a skip logic such that questions were only asked of participants to whom they pertain. For example, if a participant stated that they had no issues with personal-care matters, they were not asked subsequent questions about it.

Prompts then specified burden and life-impact categories impacted by DMD (“aided questions”), and queried further description of the impact. These questions with domains specifying aspects of DMD impact (i.e., burden) and life-impact categories summarizing life domains known to be affected by DMD in the medical research literature, and with input from clinical experts (LL, NM), DMD patients and caregivers, representatives from DMD patient organizations, and Engage Health.

### Measures

In addition to the qualitative measures described above as part of the interview, this study also used the following quantitative measures to assess ambulatory status, and demographic / clinical characteristics.

*Self-Assessment of Ambulatory Status* was assessed using the Lowes Lab Ambulatory Status Algorithm [31] (LLASA), an unvalidated clinician-derived algorithm that is used in clinical practice. This categorization utilizes a branching logic to identify the questions appropriate to the person with DMD’s level of disability. Respondents are asked three to five questions in order to categorize the person with DMD as either ambulatory, transitional, or non-ambulatory.^5^

*Demographic / clinical characteristics* included age of the person with DMD, gender, race/ethnicity, state of residence, education level of the patient and their mother. Family socioeconomic status was captured by whether the family had a computer at home, a car or van at home, the option of a free lunch at school, and whether they owned or rented their home [32]. Clinical characteristics included use of steroids for DMD and participation in DMD clinical trials.

### Statistical analysis

Two independent raters (SJ, DS) from different organizations coded the qualitative data according to a coding guide, which also included instructions for resolving differences. While the coding guide included functional-activity categories that reflected the research literature and consultations with DMD experts, the coders were explicitly tasked with also identifying new categories that reflected participant responses. Responses were analyzed separately by ambulatory status category: Ambulatory (capable of walking); Transitional (when ambulation becomes a problem, and the child requires assistance); and Non-Ambulatory (incapable of walking, wheelchair dependent). Descriptive analyses summarized participant responses to the aided and un-aided questions as a function of ambulation category. The Kruskall Wallis non-parametric rank test [33] compared ambulation group responses. This statistic is used for comparing two or more independent samples of equal or different sample sizes, and it is the non-parametric equivalent of the one-way analysis of variance (ANOVA). Non-parametric tests are useful with relatively small sample sizes, which may not have normal distributions and thus may violate assumptions of parametric tests.

## Results

### Sample

Table 1 displays the demographic and clinical characteristics of the study sample. The study sample reflected the perspective of minor and adult patients and their caregivers. Table 1 provides a breakdown of patient/caregiver groupings within each ambulation category. Since the focus of investigation was the DMD patient regardless of the source (i.e., patient or caregiver), results will be described across sources in terms of the impact on the DMD patient.

**Table 1:** Descriptive Statistics of Study Participants (n = 60)

Characteristic	
Mean Age of person with DMD (SD)	12.3 (6.1)
Age range (minimum, maximum)	3, 33

		N	%
Participant Role within Lowes Lab Algorithm Characterization (n)			
Ambulatory	20		33
Minor patient *with* parent		2	
Parent of minor patient *alone*		18	
Transitional	20		33
Minor patient *with* parent		2	
Parent of minor patient *alone*		17	
Parent of adult patient *alone*		1	
Non-Ambulatory	20		33
Minor patient *with* parent		4	
Parent of minor patient *alone*		7	
Parent of adult patient *alone*		4	
Parent of adult patient *together*		1	
Adult patient *alone*		4	
*Gender*			
Patient Gender* (no. males)		4	100
Caregiver Gender* (n)			
Male		11	18
Female		45	75
DMD Patient Race (n)			
Black		3	5
White		52	87
Asian/Pacific Islander		2	3
Other		3	5
DMD Patient Hispanic Ethnicity (n)			
Yes		2	3
No		58	97
DMD Patient Level of Education (n)			
Preschool		5	8
Currently or Completed Elementary School		35	58
Currently or Completed Middle School		9	15
Currently or Completed High School		4	7
Some college		3	5
Technical (Vocational) degree		1	2
4-year University degree (Bachelors degree)		3	5
DMD Mother's Level of Education (n)			
High School or less		15	25
Some college		15	25
4-year University degree (Bachelors degree)		23	38
Masters degree		6	10
Doctoral degree		1	2
Own computer at home (no. yes)		59	98
Car or van at home (no. yes)		56	93
Option of free lunch at school (no. yes)		20	33
Own or rent home (no. own)		42	70
*Clinical Characteristics*			
Use of Long-Term Steroids (n)			
Never		9	15
Used in the Past		6	10
Currently Use		45	75
Participation in Clinical Trials (n)			
Never		32	53
Participated in the Past		14	23
Currently Participate		14	23

*Biological sex

*Patient demographics* DMD patients in the sample had a mean age of 12.3 years (SD = 6.1). Study participants lived throughout the United States, with greater representation on the East, West, and Southern coasts. The sample was predominantly white (87%), non-Hispanic (97%), and the median current education level was Elementary School.

*Caregiver demographics* Caregivers were predominantly female, and the median level of education was some college. Almost all participants reported owning a computer in the home (99%), and having a car or van (93%). Most participants owned their own home (70%), and about one third reported having the option of a free lunch at school.

*Clinical characteristics* One third of the DMD patients described in the study sample was ambulatory, one third was transitional, and one third non-ambulatory (n = 20 per group). The age ranges for each Lowes-Algorithm stage were 3–14 years of age for ambulatory, 6–17 years of age for transitional, and 10–33 years of age for non-ambulatory. Seventy-five percent of the people with DMD currently used long-term steroids, while 10% had used them in the past but not currently, and 15% had never used them. While over half (53%) of the people with DMD had never participated in a clinical trial, about a quarter of the sample was currently in a trial and a quarter had participated in the past. The current trial participants were primarily transitional patients (n = 7 of 14).

### Treatment goals by ambulation group

Treatment goals were derived from participant answers to the following question about burden of disease: "What is the most important thing that you wish you/your child could do but cannot because of Duchenne?" The following categories of patient-centered treatment goals were built on the research literature and clinical experts: daily functioning, sports/recreation, personal care, travel/transportation, communication, relationships, employment, healthcare needs, and education. Additionally, coders identified a new category “Health,” reflecting stamina, muscles aches, and concerns about longevity.

**Fig. 1:**
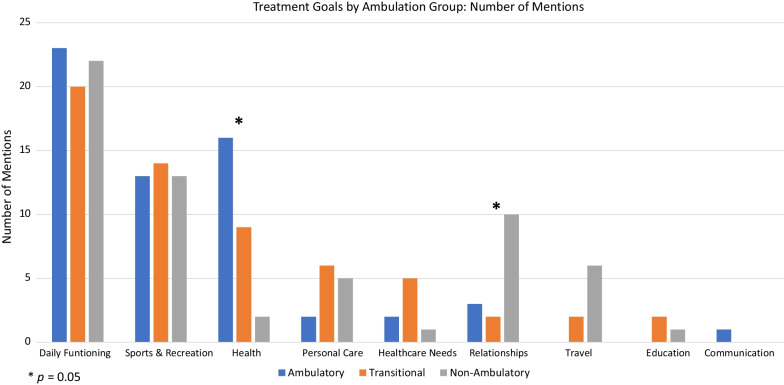
Treatment Goals by Ambulation Group: Number of Mentions (unaided and aided responses)

As patients progressed in disability, there were differences in functional areas deemed most important. While daily functioning and sports/recreation remained the most important priority areas across ambulation groups, there were notable differences in the stated importance of health and relationships (Kruskall Wallis H = 12.24 and 5.28, p = 0.002 and 0.02, respectively). Specifically, health became less prominent as the disability progressed from ambulatory to transitional to non-ambulatory phases of disability; whereas relationships became more prominent as one progressed to the non-ambulatory phase from the ambulatory or transitional phases (Fig. 1). Other indicators of differences across groups were revealed by some categories only being mentioned for transitional and non-ambulatory patients (i.e., travel, education) and another only being mentioned by ambulatory patients (i.e., communication).

### Reasons for treatment goals by ambulation group

The reasons underlying the importance of the abovementioned functional categories was addressed with the question "Why is this thing important to you/your child?” Potential categories for content coding built on the research literature and clinical experts. These reasons included: self-esteem/self-confidence, connection with others, financial situation, time commitment, and independence. Additionally, coders identified two new categories referred to as “Accessibility” and “Enjoyment.” The former reflected being able to get into places, and from one place to the next. The latter reflected enjoying life and relishing the experience.

**Fig. 2:**
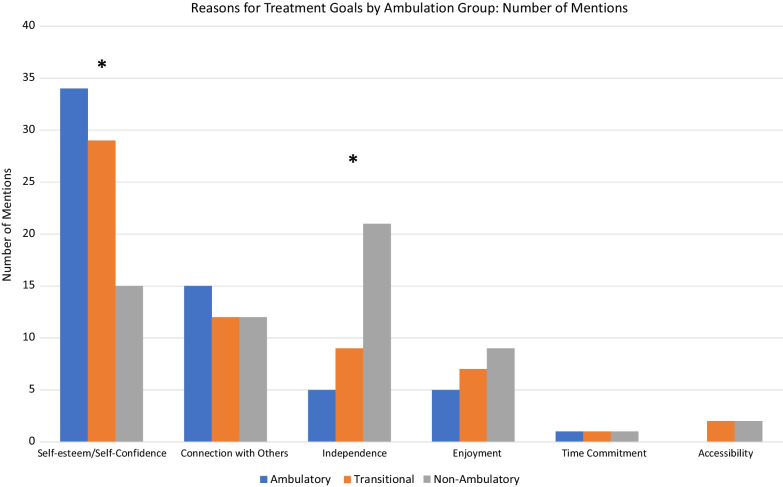
Reasons for Treatment Goals by Ambulation Group: Number of Mentions

As patients progressed in disability, the reasons underlying the importance of a particular functional domain differed across ambulation groups (Fig. 2). There were notable differences in the prominence of self-esteem/confidence and independence (Kruskall Wallis H = 9.46 and 7.35, p = 0.009 and 0.025, respectively). Specifically, self-esteem / confidence was most important for ambulatory patients, and became less prominent for patients in the transitional and non-ambulatory phases of disability. In contrast, independence was less important for ambulatory patients, and became increasing prominent for patients in the transitional and non-ambulatory phases of disability. There were, however, similarities in the importance of connection with others and enjoyment across ambulation groups.

For the domains where there were ambulation-group differences in importance as noted above, the content of the reasons was somewhat distinct. Table 2 provides examples of interview content by ambulation group. For the self-esteem/confidence domain, ambulatory and transitional patients were more focused on fitting in and not feeling different, whereas non-ambulatory patients were more concerned about feeling restricted. For the independence domain, the content for ambulatory patients exemplified feeling different from others and being motivated by independence. Among people in the transitional category, independence was more related to the challenges of functioning in a school environment and worry for the child. Once non-ambulatory, the focus of independence was related to dependence on ventilators, waiting for others to do something necessary, and the decreasing motivation to even try to do something independently because it was so difficult.

**Table 2:** Reason-Domain Content Differences by Ambulation group*

Reason Domain	Ambulatory	Transitional	Non-Ambulatory
Self-Esteem/Self-Confidence	“It is important for his self-esteem to feel like he fits in with the crowd”	“It is hard for him and the whole family when he notices he is different.”	“Just to be like everybody else, not feel so restricted”
Connection with Others	“Every kid at that age needs to have friends, it is important not to feel lonely”	“It is important for him to be a part of us together, being a out in the community, having that identity.”	“It is important for anybody to know somebody loves and cares about you”
	“Socially it is important because when kids see somebody who is weaker they tend to bully”	“Being able to fit in, to socialize, to feel that he matters.”	
	“It is harder to maintain friendships for him, it is harder to get involved”		
Independence	“Peers his age walk to school by themselves and go to the mall- he can’t do that”	“Just the independence- having to rely on others, especially at school with the bathroom”	“I would like to not have to wait for someone to help or do it for me”
	“Independence gives him the motivation to get up- I think about the effect it has on his mentality”	“To have his independence”	“Going and doing something all by himself is such a pain in the butt, he doesn’t do it”
		“Having that level of independence- we constantly worry that he will slip and fall, sense of high alert.”	I would just like to be less dependent on the ventilator, not be short of breath
		it feels like he is losing his independence, asks for someone else for help- always rely on someone	
Accessibility		“Their aunty is like their best friend but we have to worry about her stairs. We always have a conversation about the stairs.”	“I am always stuck in the same place- want to see new things and experience different types of weather”
		“He can’t get upstairs, can’t get to the bedrooms- we can’t make our entire home accessible”	“Even [now], places aren’t handicap accessible and he misses out on activities”
Enjoyment	“His happiness is so important and we want to impact his ability to have happiness”	“There is a sense of accomplishment when he achieves what he set out to do.”	“It gives him something to look forward to, usually has nothing to look forward to”
Time Commitment	“I would like for him not to miss school so much”	“He feels like he misses out a lot with his weekly infusions- you have to work around it.”	“He misses a lot of school”

*Underlined text highlights the content specifically relevant to the Reason Domain

In contrast, for domains with similar importance across ambulation groups, the content was relatively similar. The domain of connection with others was related to making and maintaining friendships in the greater community. The domain of enjoyment was related to the importance of being able to be happy, to have goals that engendered a sense of accomplishment, and having something to look forward to. The domain of time commitment reflected how much DMD treatments and doctor’s appointments impacted the patient’s participation in school and other normal activities. The domain of accessibility, which was not mentioned among ambulatory patients, was reflective of similar concerns for transitional and non-ambulatory patients: physical access to different parts of their environment. As they became non-ambulatory, this content reflected a frustration with being stuck in the same place and missing out on desirable activities.

### Worst days vs. best days by ambulation group

**Fig. 3:**
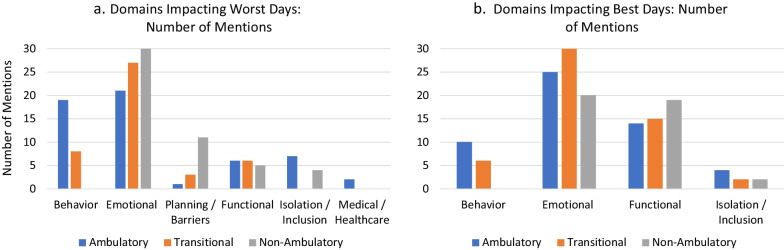
Domains impacting worst days and best days: number of mentions

Figure 3 display results of queries about life domains that impact the person with DMD’s worst and best days, respectively. It is notable that emotional functioning (e.g., sadness, anger, low self-esteem, etc.) is most prominent for all ambulation groups for both best and worst days. Functional aspects impact best days across groups as well. Behavioral issues (e.g., aggressive, prone to meltdowns, uncooperative, etc.) are most prominent for ambulatory patients’ worst days, and only somewhat notable for transitional patients. For non-ambulatory patients, behavioral issues were not at all pertinent. More domains overall were noted as having an impact on worst days compared to best days.

### Goals for new DMD treatments

**Fig. 4:**
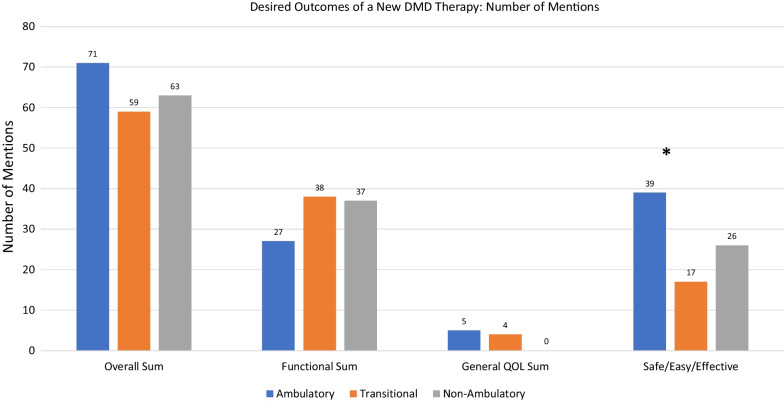
Desired DMD treatment outcomes: number of mentions

Figure 4 displays the results related to desired goals for a new DMD therapy. In addition to displaying the overall sum of outcomes mentioned by ambulation category, this figure shows the number of mentions of specific functional goals, general QOL goals, and concerns about safety, ease of use, and effectiveness. Functional goals were multidimensional, focusing on improving or maintaining muscle function and strength, organ function, independence, communication and/or cognition, stability, and energy.

Concerns about safety related to tolerability, such as avoiding issues related to long-term steroid use (e.g., immunosuppression, bone loss, emotional volatility, weight gain [34]). Tolerability also referred to concerns about sudden death, bone loss, cataracts, and pain. Ease of use related to convenience and schedule of dosing so that the treatment had minimal interference with daily life. Access reflected affordability, how soon it would be available, having a broad label, and frequency and distance of travel required to utilize a potential therapy. Effectiveness referred to the direct biological effects of the drug, such as desiring that it produced the dystrophin protein, led to metabolic change, increased bone density, reduced pain, increased growth rate, impacted lifespan, related to finding a cure, and that the preclinical results led to real clinical impact.

When asked about desired outcomes of DMD drug therapies, participants in the three ambulation groups noted similar numbers of endpoints related to functional and general QOL concerns (Kruskall Wallis H = 2.77, 5.07, and 4.83, respectively; p = 0.25, 0.8, and 0.09, respectively).There were, however, group differences in the number of mentions of concerns about safety/ease/effectiveness, with ambulatory patients ranking this concern higher than non-ambulatory and transitional patients (Kruskall Wallis H = 13.44, p = 0.001).

## Discussion

The present study provides useful information about treatment goals for DMD from the perspective of key stakeholders: patients and their caregivers. It highlights some consistent values across the ambulation disability trajectory, as well as introducing an evolution of priorities as the person with DMD becomes more disabled in ambulation. The breakdown of results by ambulation disability was an explicit choice to help elucidate how treatment goals change over ambulation disability progression. It does not invalidate other aspects of disease progression.

For example, daily functioning and recreation remain important for all patients, while relationships become a more prominent focus as disability progresses. This finding may reflect both adaptation and changing priorities. Non-ambulatory patients/parents have had more time to cope with and thus to adapt to realities such as not being able to play sports. At the same time, they may be increasingly aware of disability-related decline in peer relationships at a time when peers without DMD are more social. This increased awareness may render the maintenance of any relationships particularly important as the disease progresses.

The underlying drivers of the DMD burden domains and their meaning also evolved over the disability trajectory. For example, while self-esteem and confidence were drivers of goals for all patients, the foci were distinct at different stages of disability. For patients earlier in the disability trajectory, the concern was more about fitting in and not feeling different, whereas later they were more related to not feeling restricted. This difference may also reflect the increased isolation and loss of independence that patients experience as their disability progresses. Early on, they may be able to participate in a mainstream, school environment whereas with increased ambulation and other disability progression, such participation becomes increasingly challenging due to problems with building accessibility or access to independent educational programs. As a result, younger patients may be more aware of how they are different from their peers whereas older patients may be habituated to this difference and be more aware of frequently feeling restricted by DMD.

These changes in values and underlying meaning of the same concept over the disability trajectory are important insights gleaned from this study. There is a substantial evidence base suggesting that when people experience changes in health, they may change their internal standards, values, and / or conceptualization of a target concept [35, 36]. While much research has documented that these “response shifts” can influence the interpretation of treatment outcomes over time, the present study highlights how treatment goals, and even the underlying meaning of a broadly stated goal, may shift over time. This insight has important implications for designing treatments at different stages of the disability trajectory. It suggests, for example, that treatments that enable patients to feel more like their peers and fit in are particularly important when patients remain ambulatory. School-based interventions aimed at teaching tolerance and inclusion may also be implicated. Later in the trajectory, desirable treatments are deemed those that are accessible and not time-consuming to take so that patients can maintain some degree of independence and maintain social relationships. The acknowledged importance of relationships with family and friends among non-ambulatory patients may reflect social isolation from peers and an appreciation for all that these people are doing for them to keep them healthy [37].

Of all of the functional domains addressed in the present study, emotional functioning was found to be central in participants’ descriptions of best and worst days. This insight may have implications for the development of behavioral interventions to help patients and caregivers to cope with the emotional challenges of DMD. Coping interventions that might be worth considering in DMD include teaching coping flexibility for patients and their caregivers [38–40] and mindfulness [41, 42].

This direct information about DMD burden domains leads to insights related to goals for new DMD treatments. They underscored the importance of maintaining and improving function, tolerability, and biological effectiveness. The domains directly noted by study participants could be useful for guiding outcome measurement for DMD clinical trials. In particular, such outcome measurement should be tailored to the patient’s disability stage with different domains reflected for ambulatory, transitional, and non-ambulatory patients.

While the present work has important advantages of addressing key concepts using content analysis qualitative data, its limitations must be acknowledged. First, the sample sizes are relatively small, which is not uncommon in qualitative research. This situation prevents most statistical analyses due to low power. This was dealt with by primarily focusing on raw counts of number of mentions, and by using non-parametric tests and doing so sparingly. Future research might create close-ended questions to address these same key concepts and implementing a larger-scale study of patients and caregivers. A second limitation relates to the use of an unvalidated algorithm for categorizing patients’ stage of ambulation disability. Future research might validate this classification scheme. Alternatively, future work might utilize other validated methods for classifying amulation status. For example, ACTIVLIM is a measure of activity limitations for patients with upper and/or lower limb impairments. The scale measures a patient's ability to perform daily activities requiring the use of the upper and/or lower limbs, whatever the strategies involved. ACTIVLIM has been validated in children (age 6–15) and in adults (age 16–80) with a neuromuscular disorder [43]. A third limitation is that we did not measure or adjust for caregiver fatigue as they answered the interview questions. If they caregivers were DMD carriers, their answers might have reflected their own feelings of fatigue and muscle weakness in addition to their perceptions of their child’s experience of these symptoms. Future research should not only track whether the maternal caregiver is a carrier, but also should track and statistically adjust for the caregiver’s personal experience of fatigue and muscle weakness when rating to their perceptions of their child’s experience, assuming adequate statistical power to do so.

## Conclusions

In summary, the present study utilized content analysis of qualitative data to highlight important domains of DMD burden, underlying reasons for this importance, and goals for new treatment. It highlights variability in these concerns across the disability trajectory, and provides a roadmap for patient-centered DMD drug and intervention development. Based on our findings, this roadmap would entail a continued biomedical treatment focus on maintaining daily functioning and recreation, and a tailored behavioral-intervention approach to managing social and emotional functioning over the course of the disease. While earlier in the disability trajectory, the interventions might focus on dealing with concerns about not fitting in and feeling different, later they would focus on reframing the restrictions caused by their disability. Helping people with DMD to master their emotional functioning would benefit both the patients themselves and their caregivers, as emotional functioning was found to be central in participants’ descriptions of best and worst days.

## Data Availability

The study data are confidential and thus not able to be shared.

## Abbreviations

DMD: Duchenne muscular dystrophy
ES: Effect size
FDA: Federal drug administration
QOL: Quality of life

## Footnotes

1 The Jett Foundation is a not-for-profit organization whose goal is to empower people and families impacted by Duchenne muscular dystrophy through the development of transformative programming, educational opportunities, and ongoing support for every stage of a Duchenne journey. See https://www.jettfoundation.org/ for more information.

2 See information about funding and steering committee for more details about patient-advocacy organizations who facilitated this study.

3 Ethical standards for research done with minor children or adults not capable of giving consent requires the consent of the parent or legal guardian and the assent of the subject [29].

4 The interested reader seeking further information about the survey interview questions is encouraged to reach out to the Jett Foundation directly: https://www.jettfoundation.org/contact/.

5 For further information about the Lowes Lab Ambulatory Status Algorithm, please contact Nationwide Children’s Hospital.
